# Common and Unique Neural Systems Underlying the Working Memory Maintenance of Emotional vs. Bodily Reactions to Affective Stimuli: The Moderating Role of Trait Emotional Awareness

**DOI:** 10.3389/fnhum.2018.00370

**Published:** 2018-09-18

**Authors:** Ryan Smith, Richard D. Lane, Anna Sanova, Anna Alkozei, Courtney Smith, William D. S. Killgore

**Affiliations:** Department of Psychiatry, University of Arizona, Tucson, AZ, United States

**Keywords:** working memory, emotion, medial prefrontal cortex (MPFC), insula, body perception, interoception, emotional awareness

## Abstract

Many leading theories suggest that the neural processes underlying the experience of one’s own emotional reactions partially overlap with those underlying bodily perception (i.e., interoception, somatosensation, and proprioception). However, the goal-directed maintenance of one’s own emotions in working memory (EWM) has not yet been compared to WM maintenance of one’s own bodily reactions (BWM). In this study, we contrasted WM maintenance of emotional vs. bodily reactions to affective stimuli in 26 healthy individuals while they underwent functional magnetic resonance imaging. Specifically, we examined the a priori hypothesis that individual differences in trait emotional awareness (tEA) would lead to greater differences between these two WM conditions within medial prefrontal cortex (MPFC). We observed that MPFC activation during EWM (relative to BWM) was positively associated with tEA. Whole-brain analyses otherwise suggested considerable similarity in the neural activation patterns associated with EWM and BWM. In conjunction with previous literature, our findings not only support a central role of body state representation/maintenance in EWM, but also suggest greater engagement of MPFC-mediated conceptualization processes during EWM in those with higher tEA.

## Introduction

According to multiple theories of emotion (James, 1894; Schachter and Singer, 1962; Valins, 1966; Damasio, 1999; Smith and Lane, 2015; Critchley and Garfinkel, 2017), one primary component of emotional experience is the perception of one’s own bodily reactions (i.e., typically in response to affective stimuli). This “embodied" view of emotional experience is supported by the results of both behavioral and neuroimaging studies. For example, behavioral evidence has shown that the self-reported intensity of emotional experience is associated with the accuracy of bodily perception (Barrett et al., 2004; Critchley et al., 2004; Wiens, 2005; Pollatos et al., 2007). Other behavioral studies have also shown that emotional experience can be influenced by both true changes in bodily arousal (Schachter and Singer, 1962) and false feedback about one’s own bodily reactions (Valins, 1966). Interestingly, recognizing emotions in others also appears to require a bodily simulation process (Niedenthal, 2007). For example, studies have shown that preventing individuals from adopting emotional facial expressions reduces facial emotion recognition ability (Oberman et al., 2007), that facial emotion recognition ability correlates significantly with interoceptive sensitivity (Terasawa et al., 2014), and that impairments in the ability to experience specific emotions and recognize those same emotions in others are related (Croker and McDonald, 2005; Surcinelli et al., 2006; Marsh and Blair, 2008; Arrais et al., 2010; Buchanan et al., 2010; Marsh et al., 2011).

With regard to neuroimaging evidence, overlapping brain activation has been demonstrated for both interoceptive and emotional experiences within the insula (Zaki et al., 2012), and several additional studies have separately demonstrated the role of this cortical region in both body perception and emotional feelings (reviewed in Craig, 2002, 2009). These findings have prompted recent neuro-cognitive theories of emotion (Smith and Lane, 2015, 2016; Panksepp et al., 2017; Smith et al., 2017b,d) to suggest that bodily feelings act as one (but not the only) important piece of perceptual evidence the brain uses to infer the conceptual identity of an emotional state (e.g., “My arms and legs feel heavy, my stomach aches, and I just lost a loved one; I must be having these sensations because I am sad”).

These theories also suggest that information about one’s own emotions would need to be held in working memory (WM) in order to adaptively inform goal-directed reflection and decision-making processes (Baddeley, 2007). This ability to maintain emotions in WM (EWM) has also recently been the topic of multiple neuroimaging studies (Waugh et al., 2014; Xin and Lei, 2015; Smith et al., 2017c, 2018b), which show substantial (but not complete) overlap with the neural activations observed during WM for exteroceptive content domains (e.g., visual/verbal). For example, available studies of self-focused EWM have highlighted both lateral frontoparietal regions (common to exteroceptive WM) as well as dorsomedial prefrontal and anterior insula (AI) regions (which appear to be more involved in EWM than in exteroceptive WM) (Waugh et al., 2014; Smith et al., 2018b).

There have also been a few studies to date examining the WM maintenance of body-related information. For example, multiple studies have demonstrated that maintaining somatotopic information in WM (tactile WM) recruits somatosensory cortical regions and frontoparietal regions (Katus et al., 2015a,b; Ku et al., 2015; Wu et al., 2018), and that tactile WM appears to have attention-based rehearsal/maintenance mechanisms distinct from those underlying visual WM (VWM) (Katus and Eimer, 2018). To our knowledge, however, no study to date has examined the ability to hold interoceptive information in WM. Studies of interoceptive attention, which might be expected to partially overlap with interoceptive WM [i.e., based on sensorimotor recruitment models (D’Esposito and Postle, 2015)], have highlighted frontoparietal regions and posterior/mid insula regions, among others (Farb et al., 2013; Simmons et al., 2013). Aside from such suggestive findings, the neural basis of interoceptive WM remains an open question.

Further, no study to date has yet compared neural activations during EWM with those during the WM maintenance of perceived bodily reactions. It is therefore unclear whether EWM processes also involve considerable overlap with the body state attention/WM processes described above. One possibility is that EWM primarily reflects the maintenance of felt bodily reactions (“bodily working memory”; BWM). A second possibility is that EWM also reflects the maintenance of conceptual representations of emotions (e.g., representations of the concept of “sadness” or “fear”). A third possibility, however, is that EWM processes differ between individuals based on stable trait factors.

One particular trait that could plausibly play such a role is “trait emotional awareness” (tEA), which in part reflects the level of conceptual complexity/differentiation one has learned to use in the emotion recognition process (Lane and Schwartz, 1987). Individuals with higher tEA are thought to more thoroughly conceptualize their emotions in fine-grained ways (e.g., “I feel a mix of sadness and anger”), whereas individuals with lower tEA instead describe emotional reactions in simpler sensorimotor terms (e.g., “I feel sick to my stomach” or “I feel like punching someone”). It therefore appears plausible to suggest that EWM in individuals with lower tEA might primarily involve the maintenance of bodily percepts, whereas individuals with higher tEA might instead maintain concept-level emotion representations in WM to a greater degree. This individual difference may also be relevant to psychopathology, as lower tEA levels have been associated with both poorer physical health and multiple psychiatric disorders (Levine et al., 1997; Berthoz et al., 2000; Bydlowski et al., 2005; Donges et al., 2005; Lackner, 2005; Subic-Wrana et al., 2005, 2007; Frewen et al., 2008; Baslet et al., 2009; Consoli et al., 2010; Moeller et al., 2014); higher tEA levels have instead been associated with a range of adaptive emotion-related traits/abilities (Lane et al., 1990, 1996, 2000; Ciarrochi et al., 2003; Barchard and Hakstian, 2004; Bréjard et al., 2012).

The present study aimed to provide further clarification on the issues described above by asking participants to engage in a WM task that required goal-directed (intentional) maintenance of emotional or bodily reactions to affective stimuli. Our primary aim was to test the hypothesis, based on a recent model of the neural basis of tEA that we have proposed (Smith et al., 2017b), that those with higher tEA would also show greater activation during EWM (relative to BWM) in a pre-defined region of the medial prefrontal cortex (MPFC). Although this is a straightforward prediction of current theories of the neural basis of tEA (Lane et al., 2015; Smith et al., 2017b), it has not yet been examined empirically.

In previous studies, this MPFC region has been found to increase in activation with greater emotion-focused attention and with higher tEA (Lane et al., 1997, 2015; Gusnard et al., 2001; Ochsner et al., 2004; Frewen et al., 2008; McRae et al., 2008; Smith et al., 2014, 2017a, 2018a); its activation has also been linked to both semantic/conceptualization processes generally [i.e., as a hub of the “default mode network” (DMN); (Binder et al., 1999, 2009; Barrett and Satpute, 2013)] as well as to emotion concept representation more specifically (Skerry and Saxe, 2015; Satpute et al., 2016; Saxe and Houlihan, 2017). Yet direct comparisons of the MPFC’s role in reflection on emotions vs. bodily sensations, or tests of tEA as a moderating factor, have not yet been carried out. Based on our model (and “sensorimotor recruitment” models of WM more generally; D’Esposito and Postle, 2015), one would expect frontoparietal “executive control network” (ECN) regions to be activated across all WM conditions (Seeley et al., 2007; Nee et al., 2013), whereas activation of other cortical regions during WM would depend on the locations of the relevant representations that are being held active via ECN-mediated top-down modulation (e.g., holding insula-/somatosensory cortex-mediated body state representations active during BWM, or holding MPFC-mediated emotion concept representations active during EWM).

As a secondary aim of the study, we also ran whole-brain analyses to further characterize the degree of overlap between EWM and BWM, with the hypothesis that both of these conditions would similarly activate the insula and other regions implicated in WM and body perception. These analyses build off of a previously published report on these data (Smith et al., 2018b) in which we contrasted EWM with a VWM condition and with a matched condition involving no WM demands. In that report we found that dorsal MPFC (DMPFC) and the AI were more active during EWM than VWM, but that, relative to the condition with no WM demands, both of these WM conditions involved suppression of ventral MPFC (VMPFC) and activation of dorsolateral prefrontal cortex (DLPFC) and related parietal cortex (ECN) regions previously implicated in the executive component of WM generally (Rottschy et al., 2012; Nee et al., 2013). Given the evidence for overlap between emotion and body state representation reviewed above, these previous results suggested the possibility that BWM would display a similar pattern of activation as we observed for EWM (i.e., activation of the ECN and insula), but perhaps without the MPFC activation linked to emotion conceptualization. However, the BWM condition was not analyzed in that report, because that report focused on a different research question (i.e., regarding whether MPFC was activated or inhibited by EWM, for which there have been previous conflicting findings) that was unrelated to BWM. In the present report we examined the BWM condition for the first time, with the hypothesis that similar AI activation would be found for the BWM condition (relative to both the VWM and the no-WM conditions) as was observed with EWM in our previous report (Smith et al., 2018b).

Based on the hypotheses described above, our specific predictions were as follows:

(1) Higher tEA will be associated with greater MPFC activation in the EWM condition relative to the BWM condition.
(2) The EWM and BWM conditions will show a similar pattern of ECN and insula activation, relative to both the VWM and no-WM conditions.

Although not a strong a priori hypothesis, we also examined the question of whether somatosensory cortices might show greater activation in BWM than in EWM, because BWM includes both interoceptive and somatosensory components and because emotion has been more closely linked to interoception in the previous studies reviewed above.

## Materials and Methods

### Participants

Twenty-six healthy adult participants (13 female; mean age = 23.12 ± 4.03 years) were recruited from the general population of Tucson, Arizona, using flyers and online advertisements. Participants had no history of psychiatric or neurological disorders, as assessed via a telephone screening questionnaire based on criteria within the *Diagnostic and Statistical Manual for Mental Disorders, 4th edition; DSM-IV-TR*. Participants provided written informed consent prior to engaging in any study-related activities. Participants also received monetary compensation for their time. The University of Arizona Institutional Review Board reviewed and approved the protocol of the present study.

### Working Memory Task

**FIGURE 1:**
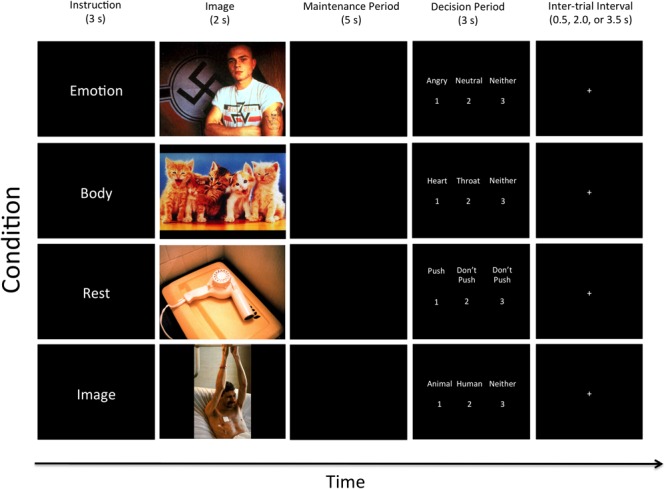
Illustration of the four task conditions. After the appearance of each instruction, an emotionally pleasant, unpleasant, or neutral image was presented followed by a maintenance period. Examples of two unpleasant, one pleasant, and one neutral image are shown here (from top to bottom). All contrasts reported in this manuscript compare the 5-s maintenance periods between the Emotion, Body, Rest, and Image conditions. The decision period that followed included making a simple identification judgment from memory that included three options (where the correct answer was different depending on the instruction associated with that condition; described in greater detail in the text). Participants did not know what condition-specific options would be presented on a given trial, but could select “Neither” if the available options on that trial were both incorrect.

After providing written informed consent, participants viewed written instructions (on a laptop computer) for the WM task (this task is illustrated in **Figure 1**). The instructions began, “you will be shown a series of pictures that typically trigger emotional reactions,” and “on each trial you will be shown one picture and given instructions to pay attention to something specific.” Participants were informed that each picture would be followed by a pause (WM maintenance period), during which only a black screen would be shown. During this pause they were to maintain the specific attended item in memory. After the pause, three options would appear on the screen, and participants were to press one of three corresponding response buttons in order to test their memory.

Trials spanning four task conditions were conducted in pseudo-random order. At the start of each trial, one of four cue words – “Emotion,” “Body,” “Image,” or “Rest” – was presented on the screen. These were explained to participants as follows:

#### Emotion

Participants were informed that the “Emotion” cue meant they “should pay attention to [their] own emotional reaction to the picture and hold this emotional feeling in mind” during the pause. Of the three options presented on the screen after the pause, two would be emotion words (including: “angry,” “disgusted,” “happy,” “neutral,” “afraid,” and “sad”), and the third would be “neither.” Participants were instructed to select (by button press) the option that best reflected the emotional response they were holding in memory.

#### Body

Participants were informed that the “Body” instruction meant they “should pay attention to [their] own physical bodily reaction to the picture and hold this bodily feeling in mind” during the pause. Of the three options presented on the screen after the pause, two would be words for areas within the body where they may have felt a change in physical sensation (including: “heart,” “stomach,” “arms,” “face,” “throat,” and “no change”), and the third would be “neither.” Participants were instructed to select (by button press) the option that best reflected their memory of the region where they felt the greatest change in bodily sensation.

#### Image

The “Image” instruction was said to indicate that participants “should pay attention to the things in the image and hold the image in mind” during the pause. Of the three options presented on the screen after the pause, two would be category words (including: “human,” “animal,” “child,” “adult,” “male,” “female,” “living,” and “non-living only”), and the third would be “neither.” Participants were to select (by button press) the option that best reflected their memory of the content of the image. This exteroceptive (visual) WM condition allowed for comparison with a more thoroughly studied WM content domain, and also allowed us to assess performance accuracy and task engagement.

#### Rest

Participants were told that the “Rest” instruction meant they “do not need to remember anything” during the pause. Of the three options presented on the screen after the pause, two would be “Don’t push,” and the third would be “Push.” They were instructed to select “Push” (by button press) on each trial. This condition served as a baseline for comparison in which nothing was intentionally held in WM during the maintenance period, but where all stimulus conditions were identical. It is important to clarify, however, that this condition could still involve a type of automatic emotion maintenance, where an affective response to a stimulus could persist during the maintenance period. However, this type of maintenance is theoretically/mechanistically distinct from goal-directed (i.e., intentional, top-down) WM processes, and these two maintenance processes have been shown to have distinct neural correlates in previous studies (e.g., see Waugh et al., 2014). Thus, this baseline comparison condition also served to remove the potential confound of automatic emotion maintenance in the analyses described below, and allowed comparisons between conditions capable of specifically highlighting the types of goal-directed WM processes typically examined in visual and auditory WM studies.

Lastly, participants were asked to employ specific strategies during the pause for each trial type. In the “Emotion” condition, they were instructed to “hold the emotional feeling in mind in order to remember what emotion it was.” In the “Body” condition, they were instructed to “hold the bodily feeling in mind in order to remember where you felt your body react.” In the “Image” condition, they were told to “hold the visual image of the picture in mind in order to remember what was in it.” The instructions also stated: “try your best to NOT simply hold a word in mind instead” in order to remember (e.g., repeating “sad, sad, sad,” “stomach, stomach, stomach,” or “animal, animal, animal”). This was done to avoid the use of an auditory/verbal WM strategy in each condition (e.g., so that participants were actually holding in mind an emotion concept in the “Emotion” condition, a visual image in the “Image” condition, etc.). After reading these instructions, participants had the opportunity to ask questions, and then practiced several trials on the laptop. This practice period allowed two exposures to each trial type. After this practice period, participants had a second opportunity to ask clarifying questions if something was still not completely understood.

Participants were then escorted to the University of Arizona’s magnetic resonance imaging (MRI) facility, where they underwent functional MRI scanning (see the section “Neuroimaging Methods”) while performing the WM task. Prior to image acquisition, they again completed several practice trials to become accustomed to performing the task in the scanner environment.

The pictures used in the task were selected from the International Affective Picture System (IAPS). On the basis of the IAPS normative data (both male/female) provided by Lang et al. (2008) using a 9-point rating scale, images were selected for each emotional valence [unpleasant (U) = *M*_valence_ < 4.0, neutral (N) = 4.0 < *M*_valence_ < 6.0, pleasant (P) = *M*_valence_ > 6.0]. The task was counterbalanced to the greatest extent possible with respect to all condition and stimulus variables. This included showing each response option a roughly equivalent number of times, as well as ensuring (1) that each task condition included the same number of pictures from each valence category (i.e., each of the four attention/memory conditions included the presentation of 10 unpleasant pictures, 5 pleasant pictures, and 5 neutral pictures in pseudo-random order), and (2) that these pictures were matched for content across conditions to the greatest extent possible.

A higher proportion of normatively unpleasant pictures was included because there is a greater number of unpleasant basic emotion categories (i.e., “sad,” “afraid,” “angry,” and “disgusted” vs. only “happy” and “neutral”). Interchanging the pictures used between the “Emotion” and “Image” conditions and the “Body” and “Rest” conditions allowed for two counterbalanced versions of the task. Participants were randomly assigned to one of the two versions to ensure that any potential influence of the different pictures seen within each condition would be accounted for within group analyses.

The entire WM task lasted 20 min, and consisted of 20 trials within each of the four conditions. The timeline of each trial was: Trial Instruction = 3 s, Image = 2 s, Maintenance Period (pause) = 5 s, Decision Period (displaying the three options) = 3 s. The decision period was followed by a variable-length inter-trial interval (displaying a crosshair), jittered to last either 0.5, 2, or 3.5 s.

### Trait Emotional Awareness

After completing scanning, participants returned to the lab and completed an online version of the Levels of Emotional Awareness Scale (LEAS) (www.eleastest.net) that employs a validated automatic scoring program (Barchard et al., 2010). The LEAS presents two to four sentence descriptions of 20 hypothetical social situations that involve both the self and one other person. The described situations are designed to elicit emotion within four categories (sadness, happiness, anger, and fear) at five levels of complexity. In the computer-administered version of the LEAS, one situation at a time is presented on the screen, followed by two questions: “How would you feel?” and “How would the other person feel?” Separate text fields are provided for responding to each question. Participants were instructed to type their responses using as much or as little space as needed to answer. The only requirement was that they use the word “feel” in their responses.

Scores for tEA level are assigned based on the words participants use in each response. The lowest possible score of 0 is given to non-feeling words. Words referring to physiological sensations (e.g., “tired”) are given a level 1 score, whereas level 2 scores are assigned to words that refer to feeling-driven actions (e.g., “punching”) and simple valence discriminations (e.g., “bad,” “good”) that have inherent avoidance- or approach-related content. Level 3 scores are assigned to single emotion concept terms (e.g., “happy,” “sad”). Level 4 scores are awarded when at least two words from level 3 are used in the same response (i.e., conveying greater emotional differentiation than either word alone). The self- and other-related responses are scored separately for each item as described above (i.e., with a value of 0–4). In addition, a “total” score is given for each of the 20 items; this score represents the higher of the self- and other-related scores unless both are level 4, in which case a total score of 5 is given for the item as long as the self- and other-related responses are differentiable from one another (for more detail, see Lane et al., 1990).

### Neuroimaging Methods

#### Image Acquisition and Processing

Magnetic resonance imaging was acquired using a 3T Siemens Skyra scanner (Siemens, Erlangen, Germany) with a 32-channel head coil. T1-weighted structural 3D MPRAGE images were acquired (TR/TE/flip angle = 2.1 s/2.33 ms/12 degree) covering 176 sagittal slices (256 × 256) with slice thickness of 1 mm (voxel size = 1 × 1 × 1). Functional T2^∗^-weighted scans were acquired over 32 transverse slices (2.5 mm thickness). The voxel size of the T2^∗^ sequence was 2.5 × 2.5 × 3.5 mm. Each volume was collected using an interleaved sequence (TR/TE/flip angle = 2.0 s/25 ms/90 degrees). The field of view (FOV) was 240 mm.

All MR image preprocessing and analysis was performed in MATLAB using SPM12 (Wellcome Department of Cognitive Neurology, London, United Kingdom^1^). Using standard algorithms, the raw functional images were realigned, unwarped, and coregistered to each subject’s MPRAGE image, normalized to Montreal Neurological Institute (MNI) coordinate space (resampled voxel size: 2 × 2 × 2 mm), and spatially smoothed to 6 mm (full-width at half maximum). The standard canonical hemodynamic response function in SPM was used. Low-frequency confounds were minimized using a 128-s high-pass filter. Serial autocorrelation was corrected using the AR(1) function. The Artifact Detection Tool (ART^2^) was used to regress out functional volumes as nuisance covariates in the first-level analysis (threshold: 3 SD in mean global intensity and volume-to-volume motion that exceeded 1.0 mm).

#### Statistical Analysis

First-level general linear models were used to contrast activation during the maintenance period between the Emotion, Body, Rest, and Image conditions for each participant. Each trial’s maintenance period was modeled as a 5-s interval, where separate first-level regressors were specified for the maintenance periods of each task condition; no trial phases other than the maintenance phase were explicitly modeled. Motion regressors (generated using ART as described above) were included in these first-level designs. The resulting contrast images were entered into second-level SPM analyses (one-sample *t*-tests) to assess the main effect of each contrast of interest across participants.

The first second-level contrast we examined was “Emotion > Body,” which should highlight all regions activated by maintaining emotions that are not also activated by maintaining bodily reactions. To test our first a priori hypothesis, the REX region-of-interest (ROI) tool^3^ was used to extract “activation values” (i.e., the first eigenvariates) in each participant from an MPFC ROI defined by a freely available atlas of regions/networks defined by correlated activation patterns^4^ (see Shirer et al., 2012). This ROI is part of the “dorsal DMN” as defined by the atlas, and is believed to play a major role in conceptualization processes (i.e., including conceptualization of bodily sensations as emotions; Binder et al., 1999, 2009; Barrett and Satpute, 2013; Skerry and Saxe, 2015; Barrett, 2017; Saxe and Houlihan, 2017). We then tested our hypothesis regarding greater distinctions between EMW and BWM (i.e., greater emotion conceptualization during EWM) in those with higher tEA by examining the correlation between LEAS total scores and MPFC activation values for the “Emotion > Body” contrast. Using a one-sample *t*-test, we also examined whether these MPFC activation values were significantly different than zero for the group as a whole (i.e., whether the MPFC tended to be more active in EWM than in BWM on average).

For whole brain analyses, we examined the “Emotion > Body” contrast along with two others: (1) “Body > Rest,” which should highlight all regions activated by maintaining bodily reactions (i.e., relative to a period involving no WM maintenance), and (2) “Body > Image,” which should highlight all regions activated by maintaining bodily reactions that are not also activated by exteroceptive WM. These contrasts, and their inverses, were analyzed in order to allow for more thorough characterization of the whole-brain similarities and differences between WM for these different content domains (i.e., bodily reactions and visual images). This builds off of a previous report from this data set (Smith et al., 2018b), which compared WM for emotions, visual images, and the rest condition, but did not investigate the Body condition (i.e., because the Body condition was unrelated to the research question addressed in that previous report). Finally, conjunction analyses were performed (within a Flexible Factorial model in SPM12) to confirm regions of activation common to (1) the “Emotion > Rest” and “Body > Rest” contrasts, and (2) the “Emotion > Image” and “Body > Image” contrasts. These conjunction analyses were performed using SPM12’s “conjunction null” function (Friston et al., 2005).

For these whole-brain analyses we first set a cluster-forming height threshold of *p* < 0.001 (uncorrected). The resulting clusters were then subjected to a cluster extent (i.e., number of voxels) threshold of *p* < 0.05 [false discovery rate (FDR) corrected]. Cluster identification/labeling was done using the automated anatomical labeling (AAL) atlas within SPM12 (Tzourio-Mazoyer et al., 2002).

## Results

### Cognitive/Behavioral Measures

As reported previously (Smith et al., 2018b), the Rest condition of the WM task had an average response accuracy of 99.0% (*SD* = 1.8%). The Image condition had an average response accuracy of 92.0% (*SD* = 7.3%). We were not able to assess accuracy within the Emotion and Body conditions, however, because there is currently no means of objectively measuring the basic emotion category or bodily reaction that a participant actually experienced. As also reported previously for this data set (Smith et al., 2018b), the mean LEAS total score was 73.7 (*SD* = 9.68).

### Correlation Between tEA and MPFC Activation

**FIGURE 2:**
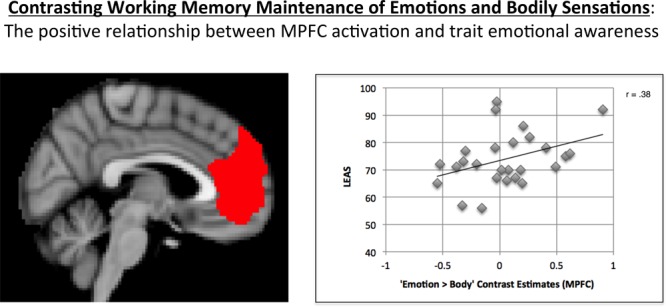
Illustration of the positive correlation observed between MPFC activation for the “Emotion > Body” contrast and LEAS scores. The MPFC ROI used is shown on the left. A scatterplot of the data is shown on the right, which illustrates the positive relationship observed.

As expected, MPFC activation in the “Emotion > Body” contrast was significantly correlated with LEAS total scores in the hypothesized direction (*r* = 0.38, *p* = 0.027, one-tailed; **Figure 2**).^5^

A one-sample *t*-test of the MPFC activation values for this contrast indicated that, across participants, the mean was not significantly different from zero (*t* = 0.76, *p* = 0.45, two-tailed).

### Whole-Brain fMRI Activation Contrasts

*Maintenance Period: Emotion > Body.* No activation clusters were observed for this contrast at our stated significance thresholds.

**FIGURE 3:**
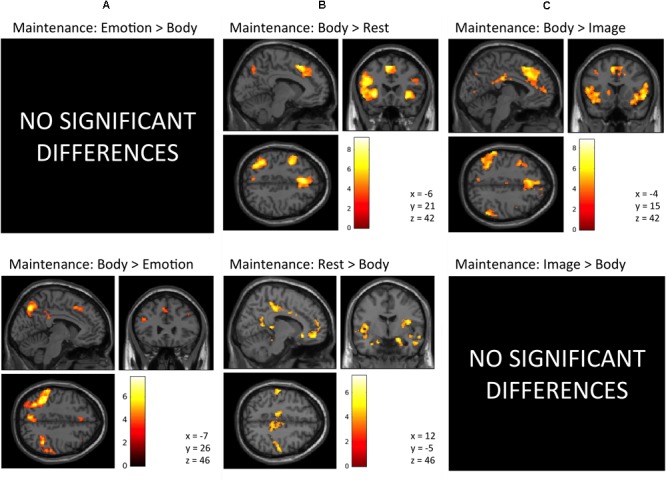
Illustration of the imaging results contrasting the maintenance period of the **(A)** Emotion and Body conditions, **(B)** Body and Rest conditions, and **(C)** the Body and Image conditions reported in **Tables 1–3**. As in the tables, the clusters displayed here are thresholded based on a cluster-forming height threshold of *p* < 0.001 (uncorrected), and a subsequent cluster extent threshold of *p* < 0.05 (FDR-corrected). Images are shown in neurological orientation (i.e., left = left; right = right). For the results of contrasts of the Emotion, Image, and Rest conditions, see our previous report (Smith et al., 2018b).

The inverse contrast (Body > Emotion) instead highlighted several clusters spanning the left and right parietal/somatosensory cortex, precuneus and posterior cingulate, DLPFC, posterior DMPFC and supplementary motor area (SMA), and other regions (for AAL atlas labels, see **Table 1** and **Figure 3A**).

**Table 1:** fMRI results: body vs. emotion.

Brain region	AAL atlas labels	Peak voxel coordinates	Cluster size (number of voxels; *k*_E_)	FDR-corrected *p*-value
**Body > Emotion (cluster forming height threshold, *p* < 0.001 uncorrected; cluster size threshold, *p* < 0.05, FDR-corrected)**				
Left parietal/somatosensory cortex	Parietal_Inf_L SupraMarginal_L Parietal_Sup_L Occipital_Mid_L Angular_L Occipital_Sup_L Postcentral_L	–58, –38, 38	1865	<0.001
Precuneus (bilateral)	Precuneus_L Precuneus_R Parietal_Sup_R Cuneus_L Parietal_Sup_L Occipital_Sup_L	–6, –68, 48	728	<0.001
Left premotor cortex	Frontal_Mid_L Frontal_Sup_L Precentral_L	–24, 10, 50	71	0.008
Right parietal cortex	Parietal_Inf_R Parietal_Sup_R SupraMarginal_R Angular_R	42, –46, 46	291	<0.001
Right DLPFC	Frontal_Mid_R Frontal_Inf_Tri_R	38, 34, 28	164	<0.001
Right DLPFC	Frontal_Mid_R Frontal_Inf_Tri_R Frontal_Mid_Orb_R Frontal_Sup_R	36, 46, 8	187	<0.001
Left posterior temporal cortex	Temporal_Mid_L Temporal_Inf_L	–54, –60, –2	193	<0.001
Left DLPFC	Frontal_Inf_Tri_L Frontal_Mid_L	–42, 28, 26	54	0.018
Right parietal/somatosensory cortex	SupraMarginal_R Parietal_Inf_R Postcentral_R	58, –38, 46	88	0.003
Right frontal pole	Frontal_Mid_R Frontal_Sup_R	26, 50, 16	126	0.001
Posterior DMPFC/SMA (bilateral)	Supp_Motor_Area_L Frontal_Sup_Medial_L Supp_Motor_Area_R	–8, 16, 48	152	<0.001
Left premotor cortex	Precentral_L Frontal_Inf_Oper_L Frontal_Mid_L Frontal_Inf_Tri_L	–44, 2, 32	181	<0.001
Right precuneus	Precuneus_R Cuneus_R	14, –66, 36	52	0.020
Left DLPFC	Frontal_Inf_Tri_L Frontal_Mid_L	–44, 50, 6	107	0.001
Posterior cingulate cortex (bilateral)	Cingulum_Mid_L Cingulum_Post_L Cingulum_Mid_R	–4, –32, 30	70	0.008
Right frontal pole	Frontal_Sup_Orb_R Frontal_Sup_R Frontal_Mid_R	24, 52, 0	40	0.040

*Maintenance Period: Body > Rest.* This contrast revealed several clusters spanning the left and right AI, DLPFC, and DMPFC/SMA, left ventrolateral PFC (VLPFC), left parietal cortex and precuneus, and other regions (**Figure 3B** and **Table 2**).

**Table 2:** fMRI results: body vs. rest.

Brain region	AAL atlas labels	Peak voxel coordinates	Cluster size (number of voxels; *k*_E_)	FDR-corrected *p*-value
**Body > Rest (cluster forming height threshold, *p* < 0.001 uncorrected; cluster size threshold, *p* < 0.05, FDR-corrected)**				
Left DLPFC/VLPFC/AI	Frontal_Mid_L Frontal_Inf_Tri_L Frontal_Inf_Orb_L Insula_L Precentral_L	–48, 26, 14	4612	<0.001
Right AI	Insula_R Frontal_Inf_Orb_R	32, 26, –2	395	<0.001
Posterior DMPFC/SMA/dACC (bilateral)	Supp_Motor_Area_L Supp_Motor_Area_R Frontal_Sup_Medial_L Frontal_Sup_Medial_R Cingulum_Mid_L Cingulum_Mid_R Cingulum_Ant_L Cingulum_Ant_R	–4, 20, 48	1045	<0.001
Left posterior parietal cortex	Parietal_Inf_L Angular_L	–38, –52, 44	956	<0.001
Left OFC	Frontal_Mid_Orb_L	–20, 40, –16	58	0.032
Left posterior lateral temporal cortex	Temporal_Mid_L	–54, –54, 4	308	<0.001
Right DLPFC	Frontal_Inf_Tri_R	42, 16, 26	104	0.004
Left precuneus	Precuneus_L	–6, –70, 46	58	0.032
Left frontal pole	Frontal_Mid_L	–28, 56, 4	53	0.037
**Rest > Body (cluster forming height threshold, *p* < 0.001 uncorrected; cluster size threshold, *p* < 0.05, FDR-corrected)**				
Right hippocampus/amygdala	Hippocampus_R ParaHippocampal_R Amygdala_R	26, –20, –16	670	<0.001
Left posterior cingulate	Cingulum_Mid_L	–16, –28, 40	229	<0.001
VMPFC (bilateral)	Cingulum_Ant_L Cingulum_Ant_R Olfactory_L Olfactory_R Frontal_Med_Orb_L Frontal_Med_Orb_R	2, 20, –8	1402	<0.001
Right posterior insula/temporo-parietal junction	Rolandic_Oper_R Insula_R Heschl_R Temporal_Sup_R	58, –36, 14	1177	<0.001
Right posterior cingulate	Cingulum_Mid_R Paracentral_Lobule_R	14, –24, 42	372	<0.001
Right posterior temporal cortex	Temporal_Inf_R Temporal_Mid_R	46, –50, 0	276	<0.001
Left Hippocampus/Parahippocampal gyrus	Hippocampus_L ParaHippocampal_L	–30, –42, –6	144	0.001
Right somatosensory cortex	Postcentral_R	56, –14, 36	385	<0.001
Right retrosplenial cingulate	Precuneus_R	10, –52, 10	192	<0.001
Left superior temporal gyrus	Temporal_Sup_L Temporal_Mid_L	–60, –14, 0	404	<0.001
Right mid-cingulate	Cingulum_Mid_R	16, 0, 32	71	0.026
Left posterior insula	Insula_L Rolandic_Oper_L Temporal_Sup_L	–48, –10, –2	424	<0.001
Left lateral occipital cortex	Occipital_Mid_L	–48, –78, 12	201	<0.001
Right anterior middle temporal gyrus	Temporal_Mid_R Temporal_Sup_R Temporal_Pole_Mid_R Temporal_Pole_Sup_R Insula_R	54, –12, –18	499	<0.001
Left somatosensory cortex	Postcentral_L Parietal_Inf_L	–56, –22, 44	145	0.001
Left hippocampus/amygdala	Hippocampus_L Amygdala_L	–30, –6, –26	161	0.001
Left caudate nucleus	Caudate_L	–18, 36, 18	124	0.002
Right posterior thalamus/retrosplenial cingulate	Cingulum_Post_R Thalamus_R	10, –32, 6	58	0.047
Right fusiform gyrus	Fusiform_R Lingual_R	28, –50, –6	65	0.034

The inverse contrast (Rest > Body) instead highlighted clusters spanning (bilaterally) the amygdala, hippocampus, VMPFC, posterior cingulate, posterior insula, and other regions (**Figure 3B** and **Table 2**). Many of these regions are known to play a role in the DMN, whose activation is typically suppressed during a goal-directed task (Buckner et al., 2008).

*Maintenance Period: Body > Image.* This contrast revealed clusters spanning regions of the left and right AI, VLPFC, DLPFC, DMPFC, SMA, ACC, posterior cingulate, parietal cortex, precuneus, and other regions (**Figure 3C** and **Table 3**).

**Table 3:** fMRI results: body vs. image.

Brain region	AAL atlas labels	Peak voxel coordinates	Cluster size (number of voxels; *k*_E_)	FDR-corrected *p*-value
**Body > Image (cluster forming height threshold, *p* < 0.001 uncorrected; cluster size threshold, *p* < 0.05, FDR-corrected)**				
Right AI/VLPFC/DLPFC Posterior DMPFC/SMA/ACC (bilateral)	Frontal_Mid_R Frontal_Sup_Medial_L Insula_R Frontal_Sup_R Frontal_Inf_Tri_R Frontal_Inf_Oper_R Frontal_Inf_Orb_R Cingulum_Ant_L Cingulum_Mid_R Supp_Motor_Area_L Frontal_Sup_Medial_R Cingulum_Mid_L Cingulum_Mid_R Supp_Motor_Area_R Rolandic_Oper_R Frontal_Mid_Orb_R Frontal_Sup_L Temporal_Pole_Sup_R	52, 20, –4	4458	<0.001
Left parietal cortex	Parietal_Inf_L SupraMarginal_L Angular_L Temporal_Mid_L Parietal_Sup_L Temporal_Sup_L	–60, –38, 38	1536	<0.001
Left AI/VLPFC/DLPFC	Frontal_Mid_L Frontal_Inf_Tri_L Insula_L Frontal_Sup_L Frontal_Inf_Oper_L Frontal_Inf_Orb_L Frontal_Mid_Orb_L Frontal_Sup_Orb_L Temporal_Pole_Sup_L Rolandic_Oper_L	–48, 26, 8	2579	<0.001
Left posterior lateral temporal cortex	Temporal_Mid_L	–48, –32, –6	107	0.002
Right parietal cortex	SupraMarginal_R Parietal_Inf_R Angular_R	52, –48, 36	390	<0.001
Left DLPFC	Frontal_Inf_Tri_L Frontal_Mid_L	–42, 28, 26	184	<0.001
Posterior cingulate cortex (bilateral)	Cingulum_Mid_L Cingulum_Mid_R Cingulum_Post_L	–4, –24, 30	313	<0.001
Left premotor cortex	Frontal_Mid_L Precentral_L	–40, 8, 44	163	<0.001
Right posterior lateral temporal cortex	Temporal_Mid_R	56, –34, –2	194	<0.001
Right premotor cortex	Frontal_Mid_R Precentral_R	42, 8, 52	64	0.017
Left lateral OFC	Frontal_Mid_Orb_L Frontal_Sup_Orb_L	–20, 38, –18	44	0.045
Left temporo-parietal junction	Temporal_Mid_L Angular_L	–42, –52, 20	68	0.014
Left precuneus	Precuneus_L	–8, –70, 38	104	0.002
Left caudate	Caudate_L	–14, 16, 2	53	0.027
Primary visual cortex	Lingual_L Calcarine_L Calcarine_R Lingual_R	0, –78, 4	59	0.020

The reverse contrast (Image > Body) instead showed no activation clusters that survived our stated significance thresholds.

**FIGURE 4:**
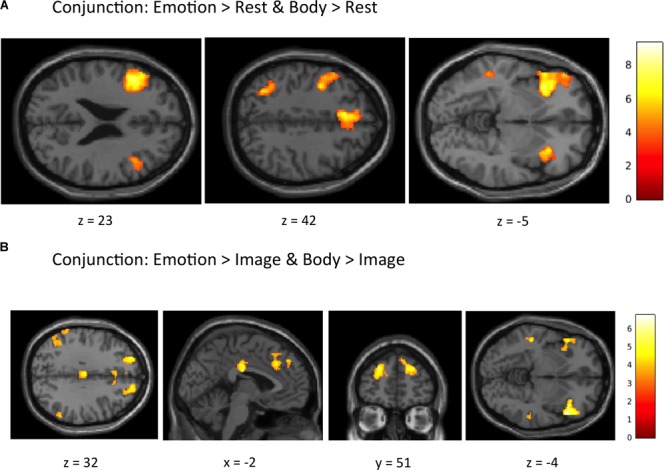
Illustration of the imaging results for conjunction analyses showing common activations within **(A)** the “Emotion > Rest” and “Body > Rest” contrasts, and **(B)** the “Emotion > Image” and “Body > Image” contrasts reported in **Table 4**. As in the tables, the clusters displayed here are thresholded based on a cluster-forming height threshold of *p* < 0.001 (uncorrected), and a subsequent cluster extent threshold of *p* < 0.05 (FDR-corrected). Images are shown in neurological orientation (i.e., left = left; right = right).

*Conjunction Analyses.* The first conjunction analysis revealed six clusters common to the “Emotion > Rest” and “Body > Rest” contrasts (**Figure 4A** and **Table 4**). These clusters spanned a set of regions including (bilaterally) the posterior DMPFC, SMA, dACC, DLPFC, VLPFC, and AI, as well as the left posterior parietal cortex and left posterior lateral temporal cortex. The second conjunction analysis revealed 11 clusters common to the “Emotion > Image” and “Body > Image” contrasts (**Figure 4B** and **Table 4**). This contrast highlights commonalities between EWM and BWM that are also significantly different from activations associated with exteroceptive (visual) WM. This analysis revealed a set of regions including (bilaterally) the AI, VLPFC, anterior PFC, DMPFC, SMA, dACC, parietal cortex, and posterior lateral temporal cortex (but notably did not include the DLPFC and other ECN regions that were similarly activated within all WM conditions).

**Table 4:** fMRI results: conjunction analyses.

Brain region	AAL atlas labels	Peak voxel coordinates	Cluster size (number of voxels; *k*_E_)	FDR-corrected *p*-value
**[Emotion > Rest] and [Body > Rest]: (cluster forming height threshold, *p* < 0.001 uncorrected; cluster size threshold, *p* < 0.05, FDR-corrected)**				
Posterior DMPFC/SMA/dACC (bilateral)	Frontal_Sup_Medial_R Supp_Motor_Area_R Cingulum_Ant_L Frontal_Sup_Medial_L Cingulum_Mid_R Supp_Motor_Area_L Cingulum_Mid_L Frontal_Sup_L	–4, 20, 48	933	<0.001
Left DLPFC/VLPFC/AI	Frontal_Inf_Orb_L Frontal_Inf_Tri_L Rolandic_Oper_L Frontal_Mid_L Frontal_Mid_Orb_L Temporal_Pole_Sup_L Precentral_L Frontal_Inf_Oper_L Insula_L	–50, 16, 12	4268	<0.001
Right AI	Insula_R Frontal_Inf_Tri_R Frontal_Inf_Orb_R	32, 26, –2	256	<0.001
Left posterior parietal cortex	Angular_L Parietal_Inf_L Parietal_Sup_L Occipital_Mid_L	–38, –54, 46	354	<0.001
Left posterior lateral temporal cortex	Temporal_Mid_L	–50, –40, –2	191	<0.001
Right DLPFC	Frontal_Inf_Tri_R Frontal_Inf_Oper_R Frontal_Mid_R	42, 16, 26	199	<0.001
**[Emotion > Image] and [Body > Image]: (cluster forming height threshold, *p* < 0.001 uncorrected; cluster size threshold, *p* < 0.05, FDR-corrected)**				
Right AI/VLPFC	Frontal_Inf_Orb_R Insula_R Frontal_Inf_Tri_R Temporal_Pole_Sup_R Frontal_Inf_Oper_R	52, 22, –4	645	<0.001
Left AI/VLPFC	Frontal_Inf_Orb_L Insula_L Temporal_Pole_Sup_L Frontal_Inf_Tri_L Frontal_Inf_Oper_L	–44, 22, 10	654	<0.001
Right anterior PFC	Frontal_Mid_R Frontal_Sup_Medial_R Frontal_Sup_R	24, 50, 30	314	<0.001
Left anterior PFC	Frontal_Sup_Medial_L Frontal_Mid_L Frontal_Sup_L	–22, 52, 30	401	<0.001
Posterior DMPFC/SMA/dACC (bilateral)	Cingulum_Ant_L Cingulum_Mid_R Cingulum_Mid_L Frontal_Sup_Medial_L Supp_Motor_Area_L	–4, 26, 36	273	<0.001
Posterior mid-cingulate cortex (bilateral)	Cingulum_Mid_L Cingulum_Mid_R	–2, –18, 30	145	0.001
Left posterior lateral temporal cortex	Temporal_Mid_L	–50, –32, –6	49	0.046
Right parietal cortex	Angular_R SupraMarginal_R Parietal_Inf_R	58, –54, 34	64	0.023
Left parietal cortex	Parietal_Inf_L SupraMarginal_L Angular_L Temporal_Mid_L	–48, –56, 30	271	<0.001
Left posterior DMPFC	Frontal_Sup_Medial_L	–4, 44, 42	54	0.037
Right posterior lateral temporal cortex	Temporal_Mid_R	56, –34, –2	69	0.020

## Discussion

In this study, we first tested the a priori hypothesis that a pre-defined MPFC region implicated in emotion concept representation (and concept representation generally) would show greater WM maintenance-related activation in the “Emotion > Body” contrast in those with higher tEA. The significant positive correlation we observed between LEAS total scores and MPFC activation in this analysis supports this hypothesis, and suggests that individuals with higher tEA may engage MPFC-mediated conceptualization processes to a greater degree when intentionally holding their own emotional responses in mind. These results confirm a straightforward prediction of recent theories of the neural basis of tEA, which suggest that engagement of the MPFC/DMN should facilitate psychological discrimination between bodily sensations that are and are not related to emotion.

These results can also be further considered in conjunction with the results of our previous report (Smith et al., 2018b). In that report we found that MPFC (and the DMN more broadly) was deactivated in the “Image > Rest” contrast, but that the majority of MPFC (i.e., excluding the most ventral regions) remained relatively more activated in the “Emotion > Image” contrast. In the context of these previous observations, our current results therefore suggest that those with lower tEA may deactivate MPFC to a similar or greater degree during EWM relative to BWM (perhaps indicating equally or more reduced levels of conceptual/semantic processing when explicitly reflecting on emotions), whereas those with higher tEA may continue to engage MPFC to a greater degree during EWM (perhaps indicating higher levels of conceptual/semantic processing when explicitly reflecting on emotions; Binder et al., 2009; Saxe and Houlihan, 2017).

These results might also be understood to suggest that, in place of abstract semantic processing, individuals with lower tEA may instead tend to reflect on their emotions using more concrete body state representations. This is consistent with a previous study (Tavares et al., 2011), which showed that, while viewing simple animated scenarios with social/emotional content, individuals with higher LEAS scores displayed greater neural activity within another abstract semantic processing region (i.e., left anterior temporal cortex), whereas individuals with lower LEAS scores displayed greater activation in a concrete action-oriented brain region (i.e., pre-motor cortex). As reviewed by Medford and Critchley (2010), many other neuroimaging studies have suggested that the AI and MPFC (particularly the anterior cingulate sub-region) are consistently activated during emotion, and that these regions may be relatively more involved in sensory and motor processes, respectively. As such, the greater participation of the MPFC we observed in individuals with higher tEA might also suggest a greater sense of agency in assessing the implications of a visceral emotional response, as opposed to passively registering the bodily sensations without considering their meaning in relation to the present situation (as may be the case in individuals with lower tEA). It is important to note, however, that greater activation of a given brain region can be interpreted in multiple ways (e.g., as indicating greater process engagement vs. indicating less processing efficiency). Thus, while our suggested interpretations here are supported by previous work demonstrating greater MPFC activation with increased semantic/conceptual processing [reviewed in Binder et al. (2009)], further work will be necessary to rule out other possible interpretations.

Our subsequent whole-brain analysis of the “Emotion > Body” contrast revealed no significant results, suggesting considerable overlap between EWM and BWM processes. This was further supported by conjunction analyses (**Table 4**), which found activations within the AI (and several other regions) that were common to EWM and BWM (i.e., relative to VWM and to the “Rest” control condition with no WM demands). These results are consistent with current theories of the neural basis of emotional experience (e.g., Barrett, 2017; Smith et al., 2017b). Such theories suggest that the MPFC (in conjunction with other regions of the DMN) plays an important role in representing the concept-level emotional meaning of bodily reactions, and that the bodily reactions themselves are instead represented in a distributed fashion across the insula and parietal cortex (among other regions). The results of the “Body > Rest” and “Body > Image” contrasts also appear consistent with such theories, because they suggest that BWM engages AI and parietal cortex (i.e., more so than passive observation or VWM). The AI regions observed in these contrasts also overlap considerably with those found in our previous report on the “Emotion > Rest” and the “Emotion > Image” contrasts (Smith et al., 2018b). This therefore also supports the overlap of emotional and interoceptive experience-related activations observed previously within the AI (Zaki et al., 2012). More generally, the whole-brain patterns of activation we observed in contrasts between the Body, Image, and Rest conditions are remarkably similar to those observed in contrasts between the Emotion, Image, and Rest conditions in our previous report (Smith et al., 2018b), supporting the idea that there is strong overlap in the overall neural processing and cognitive control (e.g., common ECN activation) of body states and emotions.

Our secondary whole-brain analyses allowed us to further characterize the neural basis of BWM. One observation of note was that the “Body > Emotion” contrast revealed bilateral parietal cortex clusters that included somatosensory cortex regions. This suggests that, while BWM and EWM both involve maintenance of insula-mediated (i.e., visceral) body state representations, BWM may involve maintenance of parietal cortex-mediated (i.e., somatosensory/proprioceptive) body state representations to a greater degree than EWM. This is consistent with the previous studies of tactile WM reviewed in the “Introduction” section above (Katus et al., 2015a,b; Ku et al., 2015; Wu et al., 2018), which have demonstrated reliable engagement of prefrontal and somatosensory cortices (but not insular cortices) during maintenance of somatotopic information. It is also broadly consistent with previous work on the neural basis of empathy for pain (Lamm et al., 2011), which has demonstrated that, while all empathic emotional experiences activate AI and anterior cingulate regions, the somatosensory cortex is only engaged when those empathic responses are triggered by directly viewing body parts in pain-promoting situations.

A second interesting observation we made was that the “Body > Image” contrast highlighted a large array of clusters spanning both medial and lateral prefrontal, parietal, and cingulate regions. Many of these areas overlap with the findings of previous studies contrasting body-focused and vision-focused attention (Farb et al., 2013; Simmons et al., 2013; Wu et al., 2018). However, this greater frontoparietal ECN activation might also suggest that the BWM condition was intrinsically more difficult/effortful than the VWM condition. This might be expected, given that continuous afferent signals from the body during the delay period may result in the need for greater interference suppression during BWM (i.e., whereas little visual interference would be expected during VWM while viewing the black screen during this delay period). This highlights one important limitation of our study that will need to be addressed in future work, perhaps via the creation/use of paradigms that allow the assessment of BWM performance differences (e.g., accuracy^6^).

More generally, however, the whole-brain analyses of BWM described above represent an important first step in answering currently open questions regarding interoceptive WM. Specifically, our results support the idea that goal-directed maintenance of interoceptive percepts involves domain-general ECN-mediated top-down maintenance signals that mediate recruitment of interoceptive cortices in the insula. These results are also consistent with similar findings regarding the neural basis of interoceptive attention (Farb et al., 2013; Simmons et al., 2013). Both of these implications can be seen as supporting sensorimotor recruitment models of WM and, more importantly, extending these models to include interoception (D’Esposito and Postle, 2015). However, it will be important for future studies to replicate these findings before they are afforded high confidence. As our BWM condition also engaged maintenance of somatotopic information (i.e., because we expected that such information might also be relevant to emotion; e.g., holding in mind a warm feeling in one’s face in response to an emotion-provoking image), it will also be important to design interoceptive WM tasks that minimize somatosensory engagement.

The present study has other limitations that are important to consider. For example, as with the Body condition, we were also unable to assess performance accuracy in the Emotion condition – because there is currently no available objective measure to assess the “correct” category of an individual’s experienced emotional state (the IAPS stimuli in our task are also known to lead to a wide variety of discrete emotions reported by different individuals; e.g., Bradley et al., 2001). Thus, while accuracy in the Rest and Image conditions was quite high (confirming task engagement), we were not able to assess individual differences in EWM or BWM capacity or the potential influence of these factors on our neuroimaging findings. This highlights the need for future studies of self-focused EWM/BWM to assess the effects of both WM load and WM manipulation (i.e., which should both increase performance demands relative to the WM maintenance of single emotions or body states in the present study). A related limitation pertains to the possible use of different WM strategies in our task. For example, although we specifically instructed participants to avoid using auditory/verbal rehearsal strategies in each condition, we cannot confirm that these instructions were followed. Future studies would therefore also benefit from the incorporation of additional measures to confirm the particular WM strategies used by each participant.

Another consideration worth mentioning is that, due to the nature of our primary research questions (i.e., regarding the neural basis of the application of WM resources to emotion- vs. bodily sensation-related content), our statistical analyses only explicitly modeled the maintenance periods of our WM task. This did not allow us to contrast the maintenance periods with the other trial phases of each condition (i.e., the instruction, image exposure, and decision periods), or to explicitly account for BOLD signal variance associated with these other trial phases. However, it is important to highlight that all other trial phases were matched across conditions (e.g., matched images and matched reading and motor response demands), such that the only differences between conditions involved the engagement of goal-directed WM resources (i.e., absent in the “Rest” condition) and the application of those resources to different contents (i.e., emotions, bodily sensations, or visual images). As such, all potential effects of these other trial phases should have canceled out in each of our contrasts, highlighting only the neural activation associated with engagement of goal-directed WM resources and their application to the specific contents of each condition. It is also worth highlighting that, according to current models (Kane and Engle, 2002; McCabe et al., 2010; Gazzaley and Nobre, 2012; D’Esposito and Postle, 2015), the engagement of WM resources should involve the same top-down modulation (or “executive attention”) mechanisms in both the presence and absence of a stimulus (i.e., involving the application of top-down amplification signals from ECN regions during stimulus exposure that can also maintain internal representations after stimulus removal). Therefore, one would expect the same top-down modulation mechanisms to be engaged during other task phases (e.g., during image exposure). That being said, with respect to EWM and BWM, this will be important to confirm in future research.

Next, it is important to note that our sample consisted of young adults with no history of psychological disorders. Thus, it is unclear whether these findings will generalize to older individuals or to psychiatric populations. It would be especially interesting to examine whether and how neural activation patterns during this task might differ in clinical disorders involving emotional pathology and somatization. In fact, the current findings are entirely consistent with a recent neural model of affective agnosia (Lane et al., 2015) that proposes a deficit in the engagement of MPFC in somatization disorders. As the MPFC is also known to regulate autonomic responses (Thayer et al., 2012), our results further highlight individual differences in a brain region that could allow individuals with higher tEA to generate/experience more differentiated and context-specific changes in emotion-related peripheral physiology (i.e., as proposed by the neurovisceral integration model; Thayer and Lane, 2000, 2009; Smith et al., 2017d). Future studies might also therefore address the possibility that higher tEA is associated with more differentiated patterns of perceived bodily responses during emotional experience (e.g., as assessed in Nummenmaa et al., 2014).

## Conclusion

At the whole-brain level, this study found that EWM and BWM were both associated with activation of a broad set of overlapping cortical regions. When combined with the results of our previous report comparing EWM to VWM (Smith et al., 2018b), these results suggest that the body state regions activated in EWM and BWM are not equally engaged during VWM, but that all three types of WM draw on a common set of ECN-mediated cognitive control processes. However, we also found evidence that individuals with higher tEA may engage MPFC emotion-conceptualization processes to a greater degree for EWM than BWM, which highlights the importance of such individual difference variables in investigating cognitive–emotional functions and also confirms an important prediction of current theories of emotional awareness (Lane et al., 2015; Smith et al., 2017b). Finally, we found that BWM activates a variety of areas previously implicated in body-focused attention. These results help clarify the distinct and overlapping neural systems underlying the interaction between bodily/emotional experience and cognitive control processes in healthy individuals, and may be useful in the future investigation of the potential breakdown of such processes in populations with emotional disorders previously shown to display lower tEA. These results also provide some initial evidence regarding a potential neural basis of interoceptive WM, a topic that has received little attention in empirical studies to date.

## Author Contributions

RS took the lead in designing and overseeing the study, analyzing the data, and writing the manuscript. RL assisted in study design and in writing the manuscript. AS assisted in data collection, analysis, and organization, as well as in writing the manuscript. AA assisted in writing the manuscript. CS assisted in data collection, analysis, and organization. WK assisted in study design and in writing the manuscript.

## Conflict of Interest Statement

The authors declare that the research was conducted in the absence of any commercial or financial relationships that could be construed as a potential conflict of interest.

## Footnotes

1 http://www.fil.ion.ucl.ac.uk/spm

2 http://www.nitrc.org/projects/artifact_detect/

3 https://www.nitrc.org/projects/rex/

4 http://findlab.stanford.edu/functional_ROIs.html

5 A *post hoc* power analysis conducted using G^∗^Power (Faul et al., 2007) revealed a power (1-β) estimate of 0.62 for this correlation analysis.

6 For example, as might be assessed by adapting current interoceptive accuracy tasks (e.g., Garfinkel et al., 2014) to incorporate a WM delay period [e.g., asking participants to attend to heart rate change in response to an affective stimulus, while simultaneously recording electrocardiography (ECG), and then assessing the accuracy of participant reports after a delay period].
